# The Treatment of Acne

**Published:** 1887-12

**Authors:** 


### *THE TREATMENT OF ACNE.*

Brocq recommends as essential in the treatment of this disease, the free use of hot water, alone or with camphor (alcoholic solution) or cologne. In addition, he gives the following formulæ, which he has found of service in his own practice:

| | | |
|---|---|---|
| ℞ | Hydrarg. bichlorid. . . . . . . . . . . . . | gr. xv. |
| | Alcohol . . . . . . . . . . . . . . . . . . | ℥iss-iii. |
| | Aquæ rosæ . . . . . . . . . . . . . . . . | ℥ivss. or iii. |

This solution may be diluted, as required, by hot water. The following is an ointment used by Hebra:

| | | |
|---|---|---|
| ℞ | Sulphur. precipitat. . . . . . . . . . . . . | |
| | Potass. bicarb. . . . . . . . . . . . . . . . | |
| | Glycerin . . . . . . . . . . . . . . . . . . | |
| | Aquæ lauro-cerasi . . . . . . . . . . . . . | |
| | Spts. vin. . . . . . . . . . . . . . . . . . . . | āā ʒii. |

This is applied during the night. On the morning following, it is removed by washing in warm water, and during the day a protective paste, like the following, is used:

| | | |
|---|---|---|
| ℞ | Zinc. oxid. pulver. . . . . . . . . . . . . . | gr. xxx. |
| | Vaselin. pur. . . . . . . . . . . . . . . . . | ℥vi¼. |

At the hospital at St. Louis, this sulphur paste is a favorite application:

| | | |
|---|---|---|
| ℞ | Sulphur. precipitat. . . . . . . . . . . . | ʒiii¾-℥viiss |
| | Alcohol. camphorat. . . . . . . . . . . | ℥viiss. |
| | Aquæ ros. . . . . . . . . . . . . . . . . . | ʒiii⅛. |
| | Aquæ destill. . . . . . . . . . . . . . . . | ℥iv⅞. |

This is also used at night, and an oxide of zinc ointment is used during the day.—*Revue Generale de Clinique et de Therapeutique.*

